# The evidence-policy divide: a ‘critical computational linguistics’ approach to the language of 18 health agency CEOs from 9 countries

**DOI:** 10.1186/1471-2458-12-932

**Published:** 2012-10-30

**Authors:** Erica Bell, Bastian M Seidel

**Affiliations:** 1 University Department of Rural Health, University of Tasmania, Private Bag 103, Hobart, TAS, 7001, Australia; 2 School of Medicine, University of Tasmania, Private Bag 34, Hobart, TAS, 7001, Australia

**Keywords:** Evidence translation, Evidence-practice divide, Evidence for policy, Local community health research

## Abstract

**Background:**

There is an emerging body of literature suggesting that the evidence-practice divide in health policy is complex and multi-factorial but less is known about the processes by which health policy-makers use evidence and their views about the specific features of useful evidence. This study aimed to contribute to understandings of how the most influential health policy-makers view useful evidence, in ways that help explore and question how the evidence-policy divide is understood and what research might be supported to help overcome this divide.

**Methods:**

A purposeful sample of 18 national and state health agency CEOs from 9 countries was obtained. Participants were interviewed using open-ended questions that asked them to define specific features of useful evidence. The analysis involved two main approaches 1)quantitative mapping of interview transcripts using Bayesian-based computational linguistics software 2)qualitative critical discourse analysis to explore the nuances of language extracts so identified.

**Results:**

The decision-making, conclusions-oriented world of policy-making is constructed separately, but not exclusively, by policy-makers from the world of research. Research is not so much devalued by them as described as too technical— yet at the same time not methodologically complex enough to engage with localised policy-making contexts. It is not that policy-makers are negative about academics or universities, it is that they struggle to find complexity-oriented methodologies for understanding their stakeholder communities and improving systems. They did not describe themselves as having a more positive role in solving this challenge than academics.

**Conclusions:**

These interviews do not support simplistic definitions of policy-makers and researchers as coming from two irreconcilable worlds. They suggest that qualitative and quantitative research is valued by policy-makers but that to be policy-relevant health research may need to focus on building complexity-oriented research methods for local community health and service development. Researchers may also need to better explain and develop the policy-relevance of large statistical generalisable research designs. Policy-makers and public health researchers wanting to serve local community needs may need to be more proactive about questioning whether the dominant definitions of research quality and the research funding levers that drive university research production are appropriately inclusive of excellence in such policy-relevant research.

## Background

Generalisable, big-*N* quantitative biomedical, rather than local community health and services research, heavily dominates health research funding programs. The USA is by far the biggest producer of that research— almost a quarter of the approximately 22 million research items in the database PUBMED are from the USA, although the USA has 4% of the total global population [1,2]. It has been estimated that the USA spends an estimated 1.5% of all health research funding on health services research [3]. The situation is similar in other developed countries, for example, the Health and Medical Research Council in Australia spends 3.8% of its funding on health services research [2,4].

However, there is growing concern about the extent to which this hierarchy of quality and the priorities it shapes for funded research translates into community benefits. A watershed report in 2001 *Crossing the Quality Chasm* by the USA Institute of Medicine suggested that the emphasis on generalisable discovery science has not translated well into innovations for community benefits and healthcare system development [5]. It has been claimed that, in most journals in the clinical sciences literature, less than 1% of published journal papers are clinically relevant [6,7]. Doubts are being raised about a too singular reliance on traditional high-powered big-*N* randomised clinical research approaches, as well as systematic reviews based on such evidence, for the multi-factorial decision-making contexts of clinical practice [6-11].

Such concerns about the translation of research into tangible benefits instigated the USA’s National Institutes of Health’s 21^st^ Century research roadmap for translational research. The developing science of ‘translational research’ for clinical practice and associated journals such as *Implementation Science* and *Translational Research* are part of a growing body of 21^st^ Century research literature on the evidence-practice divide [11]. ‘Translational science’ has taken a strong ‘laboratory bench to clinical bedside’ (‘T1’) focus, as well as ‘clinical research to clinical practice’ (‘T2’) focus [1,12,13]. Concerns about evidence translation have also led to the ‘comparative effectiveness research’ (CER) movement, initially supported by a number of influential agencies such as the USA’s Institute of Medicine, National Institutes of Health, and the Agency for Healthcare Research and Quality, developing more systematic evidence for understanding intervention effectiveness in ways that inform practice and policy [14].

However, questions about the social usefulness of evidence that has a too singular reliance on narrow biomedical research paradigms—high-powered quantitative randomised clinical trial designs—are certainly not restricted to medicine. In public health research, influential voices such as Evans and Stoddart have argued that an engagement with the contextual evidence on the social determinants of health—most useful to addressing the true underlying causal factors at work in unequal health outcomes—are not well served by narrow biomedical research paradigms [15,16]. The large body of literature on the social determinants of health, including for rural and traditional indigenous societies, points to the local and contextual socio economic shapers of health, including health literacy and health behaviours shaped by culture [17-25].

More broadly, popular and scholarly critiques of the relevance of research have suggested that, in the late 20^th^ and early 21^st^ Centuries, quantitatively sophisticated research has come to be associated with undemocratic styles of policy-making serving narrow economic interests [26,27]. These arguments have suggested that quantitative research has been increasingly used as an instrument of policy-making practices that are removed from the wider interests of society [26,27]. For example, a too singular reliance or ‘trust in numbers’ has been seen by historians of research methods as part of a historical shift in modern policy-making towards techniques of power that are (paradoxically) less rather than more accountable—distancing evidence from the experiences and views of local communities [28]. In such arguments, rightly or wrongly, big-*N* statistical research methods are seen as instrumental to the creation of a culture of mistrust of research that may itself be part of the research translation divide [26,27].

There is also a growing body of literature suggesting that the evidence-policy divide in health is complex and multi-factorial. The developing discipline of health policy studies has been led by Davies and Nutley [29] in the United Kingdom where much of the drive of ‘modernising’ the public service originated with the Blair government which produced a wide range of initiatives aimed at developing evidence-based policy-making. Drawing on the work of Weiss, Davies and Nutley suggest the complexity of evidence take-up. They describe different ways in which evidence can be used in policy-making, ranging from rational and deliberative, to strategic and political, to justifying a pre-determined position, to using evidence as a delaying tactic [29,30].

Accordingly, the translational challenge in policy can be understood in terms of multiple lines of ‘two-way’ translation from policy-makers to their communities of interest (stakeholder groups) and vice versa. It can also be understood in terms of evidence translation from policy-makers to health services, evidence translation across different kinds of health services, and evidence translation from health services to practitioners and ultimately patients. This study is an analysis of what research methods policy-makers perceive as useful: sometimes this involves focussing on the health-policy divide while other times it involves a focus on wider issues of research-policy-practice translation.

Over the last decade in particular, the barriers to research translation in health policy have been understood as being about a wide range of factors such as the inherently political nature of policy-making [31], the differing strategic priorities of policy [32], the difficulty of capturing different stakeholder interests that lie outside ‘best practice evidence’ [33], and the ‘real world’ contextual constraints of policy decision-making [34]. The ‘two worlds’ view of the evidence-policy divide—that policy-makers are from Mars and researchers are from Venus—has appeared to dominate ways of understanding the evidence-policy divide [35].

However, some policy researchers and social scientists have argued that the evidence-practice divide is also about how traditional quantitative research methods sometimes do not translate well to policy because they have certain homogenising and simplifying tendencies [36]. This is essentially an argument that the evidence-policy divide is also about the technical limitations of big-*N* statistical research methods [33,37]. This is, of course, a criticism that can arguably be made of many research approaches, including qualitative methods. In such arguments though, technical aspects of statistical evidence are seen as having an apparent complexity to lay audiences while also simplifying or homogenising complex causality with ‘correlational thinking’ [38]. A related argument in some health policy literature is the suggestion that there is a mismatch between the generalisability focus of biomedical and epidemiological research and the local community and service development needs of policy-makers i.e. that this mismatch has also helped widen the divide [39-41]. Such arguments do align to some extent with sophisticated contemporary discussion by luminaries such as Poole, Goodman and Ioannidis in debates in the clinical methods and epidemiology literature about the simplistic and widespread use of *p-*values to interpret data [42-44]. Thus, arguments about certain features of big-*N* quantitative methods as themselves representing a barrier to overcoming the evidence-policy divide have focussed on the simplifying nature of *p-*values and the perceived failure of big-*N* high powered studies to capture small-*N* rich local contexts.

Are such arguments accurate representations of the evidence-practice divide, even partly? Further exploration of this issue is important to those who are interested in learning about what researchers can do to develop more policy-relevant approaches. Clearly, a number of studies are needed to empirically validate such views, including data on policy-makers’ perceptions of the evidence-policy divide. However, to date there has not been a study exploring whether and how the most senior policy-makers might agree with this argument i.e. that the evidence-practice divide is to some extent an artefact of specific limitations of research methods. Senior policy-makers—such as CEOs of major national and provincial health agencies—could be viewed as critical witnesses and shapers of the interface between health systems and political decision-making and of evidence and policy.

This study aimed to explore whether the most influential policy-makers see a research-policy divide and if so, whether they see particular research methods as part of the challenge of bridging the evidence-policy divide. In particular, the study aimed to better understand whether and how policy-makers described particular research methods as less or more able to engage with their local contextual needs—and whether they perceive the methodological challenge of capturing local context as part of the evidence-policy divide.

## Methods

This study combines two methods—one novel and based on computational linguistics and a second using traditional critical discourse analyses. Therefore, it is termed a ‘critical computational linguistics’ approach. In summary, a purposeful, pragmatic sample of 18 national and state health agency CEOs from 9 countries was obtained. Participants were interviewed using open-ended questions that asked them to define specific features of useful evidence. The analysis involved two main approaches 1) quantitative mapping of interview transcripts using Bayesian-based computational linguistics software to measure the presence and relative frequency of co-occurrence of key concepts and 2) qualitative critical discourse analysis to explore the nuances of language extracts so identified.

### The study sample and interview procedure

The participants were recruited by direct email and telephone to leading government health agencies in OECD countries, as well as health agencies in two health systems that could provide a Chinese and Asian perspective and met the criteria of publishing at least some of their policy documents in English (Hong Kong and Singapore). In the case of the USA and the UK, regional and state health agency CEOs were also approached, as these two countries disproportionately lead evidence production in the developed world. The study used a ‘first past the post’ approach in which no further recruitment was undertaken once 18 health CEO interviews had been obtained (we judged this number to be sufficient for our purposes and the maximum resources allowed). The 18 interviews comprised 5 interviews from the USA, 4 from the UK, 2 from Australia, and one each from New Zealand, Canada, Norway, Denmark, Singapore, and Hong Kong. The response rate to the initial approach to policy-makers was about 50% i.e. about 36 agencies had been approached by the time 18 participants were recruited. Interviews were conducted from March to May 2009.

Accordingly, the recruitment method maximised the chances of obtaining policy-makers who were most interested in research translation issues and/or had a particular interest in communicating their views about such issues to research communities. The names of health agencies cannot be supplied as a condition of our ethics committee approval, as this would immediately identify their CEOs. However, all agencies shared the criteria of being leading government health agencies directly involved in delivering healthcare and developing policy. The CEOs interviewed had a major role in deciding health system policy in their country and operated at the interface of that health system and political decision-making in their country.

Interviews were of 45–60 minutes in length and were conducted by EB using open-ended questions, sent to policy-makers beforehand, which asked policy-makers to define specific features of useful evidence. Interviews were prefaced by advice designed to encourage policy-makers to view themselves as experts in the research methods needed for their contexts:

"Senior health policy decision-makers are accustomed to examining a wide range of information and research evidence for that decision-making. Many of them have high level qualifications, including research qualifications and experience. They are also likely to have experience recruiting, as well as managing or otherwise advising, researchers for health policy. Thus they have formed views about the kinds of research and ways of doing research that work best for health policy decision-making. The interview questions below ask you to use your professional judgment and experience in this area as a health policy decision-maker to offer practical advice to researchers about how to better meet your needs."

The interview questions were designed to focus the attention of policy-makers on specific features of research evidence important to understanding the methodological barriers to research-policy translation. The questions therefore focussed on research practice from conceptualisation of the aims of research to the writing of conclusions and the dissemination of research. The questions were asked sequentially, as follows:

1. Can you give some practical examples of how decisions are made about policy—what influences these decisions? What role does research have in policy-making?

2. Can you describe the features of a research report that really met the needs of health policy decision-making? What was it about that report that worked for health policy decision-making?

3. What’s the best way for researchers to find out about the policy challenges they need to address in their research?

4. What do researchers need to cover when they do literature reviews for policy decision-makers?

5. What practical advice can you give to researchers about how to make their research methods more relevant to your needs?

6. How should researchers work with community stakeholders to make their research effective for policy-makers?

7. Can you describe the findings of data analyses (graphs, tables, narrative analyses etc.) that have/haven’t worked for the practical needs of policy decision-making?

8. How should researchers write health policy options and recommendations that work for you?

9. What practical tips would you give researchers to help them make sure their research doesn’t sit on a shelf gathering dust, but rather gets used by policy-makers and their communities?

Interviews were conducted by telephone, audiotaped and transcribed for analysis.

### Analytic procedure

The analysis of the transcribed interviews involved two main stages.

#### Stage 1

*Use of language analysis software* known as Leximancer (version 4) [45] to quantify and display the conceptual structure of the interviews: the presence of key concepts, as well as the frequency of co-occurrence of key concepts (or main relationships between key concepts) in the interview transcripts. The Leximancer program is used in this study as a quantitative tool for a first stage content analysis to avoid the analyst fixating on singular concepts or terms and help reduce researcher bias. It draws on approaches in computational linguistics and has been described by its designers in their validity evaluation study [46] in following manner. The software is a highly iterative numerical model that operates to render large amounts of language data into a complex network system. In simple terms, it automatically selects a ranked list of key words in a set of documents on the basis of their frequency and co-occurrence i.e. use together. The program then builds a thesaurus which extends the seed words into weighted terms called concepts. The text is classified into text blocks (normally of a few sentences) to produce a concept index and a concept co-occurrence matrix. The software then calculates the relative co-occurrence frequencies of the concepts to obtain an asymmetric co-occurrence matrix. The matrix is used to produce a concept map informed by a clustering algorithm [46].

We believe that the whole field of computational linguistics offers much to the empirically minded health sciences. Readers are referred to the designer’s validity evaluation for more technical information on the Bayesian decision-theory design of the software [46]. Thirty-six studies in the database SCOPUS refer to Leximancer in their abstract or title and key words, with 8 of these in the health sciences, mostly studies of doctor and patient perspectives gathered from qualitative data [47-54].

The use of the software in this study involved selection of key concept words by the researcher. The key concept words were designed to be words that were meaningful descriptors of both the processes of policy-making and also of research. The key concept words were all those research and policy words in a list of common words used by policy-makers in the transcripts, as identified by the software:

•research concept words were : research, academic, methods, evaluation, analysis, literature, quantitative, qualitative

•policy concept words were: policy, policy-makers, implementation, consensus, options, improvement, decision.

The Leximancer program was used to ‘heat-map’ these key research and policy concepts as dark red to bluish spheres on the output map the software provides (different algorithms are available for clustering the concepts, however, a simple linear algorithm was selected for this study). As the concept map is stochastic (it can appear slightly differently depending on the fuzziness of words though the fundamental relativities remain unchanged), the concept map procedure was repeated 10 times.

The study also involved a separate subsidiary analysis of ‘sentiment words’ associated with ‘people types’ in the interviews. This was designed to explore further whether common terms used by policy-makers in referring to different social groups were differently associated with negative or positive sentiment words (in the extensive Leximancer thesaurus). Thus, the analysis involved mapping, in a separate quadrant table, the presence and relative frequency of co-occurrence (with sentiment words) of the following ‘people type’ words in the language of policy-makers: policy-makers, university, academic, scientists, patient, practitioners, clinicians, legislator, politicians, stakeholders, community, society, people.

A critical feature of Leximancer is that it allows the researcher to access all language data selected by the software to form the basis for the software output. This is made possible through the interactive nature of the software which presents the researcher with multiple windows through which language data can be scrutinised. Accordingly, the researcher scrutinised all text blocks selected by Leximancer for each of the key concepts used, in the context of the original interview transcripts. The researcher also manually scrutinised all text blocks selected by the software for the quadrant table, associated with negative or positive terms. In short, the Leximancer procedures were conducted in an interactive, iterative fashion that involved scrutiny of the original dataset through Leximancer’s data windows, and cross-checking of the validity of the concepts used.

#### Stage two

*Qualitative analysis* of extracts selected by Leximancer as characteristic of key concepts. The qualitative analysis was framed by critical discourse analysis to allow us to move beyond simple description of content to examining how the language of policy-makers worked in more subtle ways in its explanations of the nature of useful research and the evidence-policy divide. Critical discourse analysis is a more traditional and well-established approach that is often associated with founding thinkers on power and language such as Fairclough [55], Foucault [56-59], Bourdieu [60-62], and accordingly a summary only is given here. It is a growing area of application in health studies with 700 discourse analyses in PUBMED, 600 in the last decade, across a wide range of health practice and policy areas [63-73]. However, critical discourse analysis has not yet been used to understand the ways health policy-makers describe the evidence-practice divide.

Critical discourse analysis involves a focus on the subtle nature in which language works to shape meaning—in this study the meanings created in discussion of the evidence-policy divide. The traditional focus of such analysis is first on establishing a global hierarchy or ‘order of discourse’ (dominant narrative forms) and, second, on analysing how language works in almost invisible ways to naturalise certain assumptions as truths that serve the interests of certain social groups [57,58,74]. We hypothesised that the extracts of interviews identified by Leximancer under the different key concepts would offer windows of opportunity for understanding how policy-makers used language to legitimate or naturalise their understandings of the evidence-policy divide. Accordingly, using critical discourse analysis in this way allowed us to explore how policy-makers are themselves meaning-makers of understandings about the evidence-policy-and-practice divide.

Our procedure in employing this key aspect of critical discourse analysis—how language works to normalise assumptions about the evidence-policy divide [56,57,74]—was as follows. For each of the key research and policy concepts used in this study, the researcher scrutinised each and every extract or ‘text block’ selected by the software as an instance of that concept (again, in the context of the original interview transcript). The researcher described the way the language in that extract worked to normalise a particular view of the evidence-practice divide. These descriptions are summarised in this paper in conjunction with the software output (the concept map and quadrant table). Our focus on the way language works to ‘normalise’ or make certain assumptions appear natural, in line with the discourse analysis literature [56,57,74], explains why we did not choose to use other methods for stage 2, such as grounded theory, which do not begin with this premise about how language works.

In summary, our method aimed to use the strengths of critical discourse analysis (analysis of the subtle nuances of language) with the strengths of software-based computational linguistics (offering greater reliability in the initial gross language scoping exercise to establish the larger hierarchy of key concepts). Our use of a ‘critical computational linguistics approach’ was designed to help overcome the acknowledged reliability concerns about critical discourse analysis while also helping deliver the richer and more nuanced discussion that content analysis can sometimes lack as an essentially descriptive approach [46,75,76]. Accordingly, our epistemological position might be described as a ‘middle road’ between the philosophical traditions that inform critical discourse analysis in which meaning is highly relative and subjective, and the positivist demands of health sector research for empirical veracity and quantification. In this epistemological framework, the production of meaning occurs in a zone of collaborative human and artificial intelligence not imagined by the founding figures of critical discourse analysis [56,77,78]. Yet our study is informed by a premise consistent with their fundamental insights i.e. that the evidence-policy divide is a social construct actively produced in the language of policy-makers.

While the first author EB conducted both the interviews and produced the first draft of the analysis, we do not believe that exposure to the context of the interviews (implicit cues such as tone of voice) could somehow bias that first draft, which was checked and refined in collaboration with the second author BS. The fundamental conclusions of the study are also founded in the machine-driven Leximancer software output. Conclusions from the qualitative analyses are documented with supporting quotations from interviews in the results section. In such an epistemological approach the goal is not to free language data entirely from interpretative constraints, but rather to deliver an interpretation that is supported by evidence triangulated through the dual methods.

Further simple manual checks (readings of the paper-based versions of the interview transcripts), were performed for all interviews to ensure that the findings so obtained had not omitted anything important or introduced anything inconsistent.

Ethical approval for the study was obtained from Tasmania’s Human Research Ethics (Tasmania) Network, in line with requirements for the conduct of research by Australia’s National Health and Medical Research Council.

## Results

### The concept map

**Figure 1:**
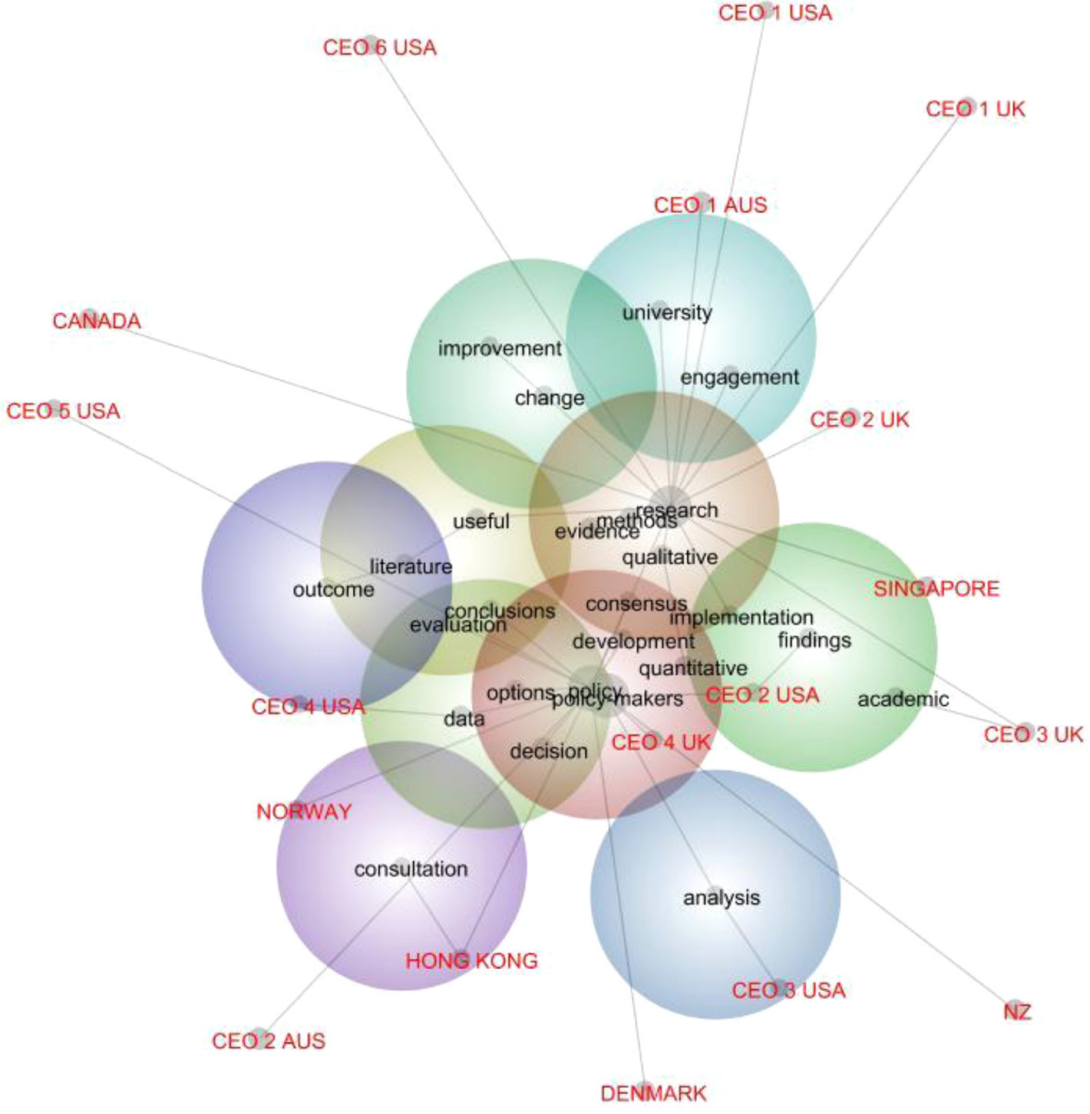
Concept map of individual CEO interviews.

Figure 1 provides a map of the key research and policy concept words using the topical (linear) clustering algorithm in Leximancer. It also shows a grey network of connections between concepts. The lines between the concepts and CEO tags show typical pathways between the concept terms in the language data. The warmth of the circles indicates the overall relative frequency of terms. The closeness of concepts indicates their contextual proximity when all other concepts are considered.

Not surprisingly, the broad concepts ‘research’, ‘policy’ and ‘policy-makers’ are most frequently found: they have a 100%, 86% and 81% likelihood respectively of being found in any one of 2,414 text blocks (of about one paragraph each) from all the interviews included in the study. In relation to paired concepts or co-occurrences, the concept of ‘research’ is most associated with methodological terms like ‘qualitative’, ‘quantitative’, ‘methods’, as well as ‘university’ which respectively have a 96%, 89%, 86% and 78% likelihood of being found in the same text block as the term ‘research’. The concept of research is least associated with terms like ‘consultation’, ‘analysis’, ‘data’, ‘literature’ (14%, 17%, 23%, 24%). In contrast, the policy concept is most associated with terms like ‘policy-makers’, ‘decision’ ‘conclusions’ and ‘development’ (99%, 88%, 80%, 64%). The policy concept is least associated with ‘qualitative’, ‘quantitative’, ‘methods’, and ‘improvement’ (21%, 19%, 18%, 11%). Thus, one broad finding is that the decision-making, conclusions-oriented world of policy-making is constructed separately, but not exclusively, from the methodologically-based world of research.

This does not mean that policy-makers discussed research in terms that suggested it was irrelevant to their needs. In fact, the policy-makers’ discourse about ‘research’ in the 569 text blocks where the term is used is not a discourse of devaluing but rather of normalising the idea of a distance between researchers and policy-makers as a function of the technical nature of research and its credibility and accessibility:

"Researchers have to keep in mind the audience that they have, and I am certainly not criticising the members of Congress in any way, but recently someone pointed out here the fact that there are virtually no economists that are members of Congress and with many of these issues the economics are critical, and so it is trying to go up to members of Congress with an elegant formula that has been modelled to perfection, but if you can’t explain it in a way that makes sense, or is going to be able to be implemented in a way that they will be able to explain to the people who they serve, ultimately it will sit on a shelf. (CEO 4 USA)"

"These people are inundated with research. They’re inundated with papers, they’re inundated with things that they need to read to understand more fully what’s going on. I think that most of the time if they feel that the research is sound that they go with it. What makes them have confidence in the research probably depends on where the research comes from, the credibility of the researcher themselves—in other words maybe Harvard would be more credible than the University of Bermuda or something. If the research is done by somebody they know—which doesn’t happen very often—that enhances the credibility. Probably who financed the research I think is incredibly important, particularly in medicine and drug company studies etc. And quite honestly how the research is presented, if it’s really good research and it’s presented in a very easy to read and understandable manner I think it’s much more credible. (CEO 6 USA)"

The discussion of ‘qualitative’ and ‘quantitative’ methods in 23 and 18 instances of uses of these terms tended to position qualitative research as non-technical and therefore more able to operate in the world of policy-making, albeit with its own limitations. Quantitative research was valued but positioned as a more specific cause of the policy-practice divide in part due to a lack of technical capital among policy-makers:

"I think we do have a problem. There are two bits to the problem; one is that in terms of health system policy, health system management, health system development, we’re sort of frightened about the quantitative type of analysis that our scientific colleagues are used to. And they are often very sceptical about the qualitative type of stuff that we would tend to do. There’s a problem there. So when we try and present policy which we say has a good evidence base to it, it’s often not respected or regarded by clinicians who in reality have to then go and implement that policy. So that does exist, and part of it is… us understanding and accepting that there is a genuine need for good solid quantitative research to take place in certain areas. (CEO AUS)"

However, the evidence-policy divide was also positioned as being about the limitations of research methodology itself. This was apparent even in the use of terms least associated with ‘research’. For example, in the 15 instances where research was discussed in the context of ‘data’, the language of policy-makers tended to describe a kind of real world information for policy-making as shaped by their own complexity-oriented intuitions about those contexts that formal research, including of community opinion, may not properly capture:

"I like to see a properly conducted survey that gets to the grass roots, that gets to the patient. That’s what would convince me: that the question is properly structured, it’s open ended, it’s not leading question and it’s asking the right people. I’m very convinced by that kind of survey which would be my number one thing. My number two would be does it feel right, is there a sort of internal logic to what’s being put forward: if I put myself in the patient’s shoes can I understand that they would feel that way…. the intuitive logic of it, does it feel right that people would be that way. If it doesn’t feel right then I’ll look more closely at their methodology and think maybe they asked leading questions and maybe they’re drawing conclusions from data that theyshouldn’t. (CEO HONG KONG)"

The role of research in policy decision-making is described typically in the 57 instances where the term ‘decision’ was used as highly fallible and susceptible to the political exigencies of that real world decision-making. Policy-makers described evidence used in policy-making as too often shaped from its inception by the need to support an outcome that has already been decided, though that was not the kind of evidence-based policy-making they preferred:

"… there’s an awful lot of research questions that manifest from ‘justify this for me’ or ‘construct it in a way to support the decision that they want to make, is going to be made’. I think that’s bad research. It does look at a specific set of issues, but it doesn’t actually help you inform policy. It’s probably motivated by some managerial, political or ad hoc approach to research. If you want to sit down and say—and this just won’t happen but, you know, ‘How should you design a perfect health care delivery system?’ and ‘What policy do you need to implement around that, around the social fabric of society, around the cultural influences, around how much you’ve got to spend, what the disease profiles are?’ You know that’s where you would really love to be, doing that and actually saying, ‘So what would be the most interesting, the most beneficial policy to come out of that?’ (CEO 2 AUS)"

The language of policy in these interviews does not so much devalue the contribution of research to ‘change’ in the 12 instances where this term occurs, as suggest that, while clinical departments do follow international research, clinical practice ideally operates on an expanding frontier of innovation that cannot always wait for sound evidence:

"Lord Darcy came to see us the other week because the government’s published a research commissioning standard and policy—but it’s too late in the year, it has already happened because good innovative centres see the horizons coming. They network clinically, internationally. They observe research that’s beginning to emerge, even when some of it is a little tenuous and crude, and build upon that and then work towards appropriate change… in a large clinical centre the role really is to keep pushing the boundaries, to continue innovating and not always wait for the well-founded evidence-based practice. (CEO 1 UK)"

Yet it is also not the case that these policy-makers positioned policy-making as being able to achieve improvement unproblematically in a non evidence-based zone. The language used by policy-makers suggested the issue is more that the dominant research methods in health don’t engage with the needs of system improvement. In the one instance where policy was associated with the term ‘improvement’, it was in a manner that positioned classical empirical health research as being poorly equipped to engage with the imperatives of system improvement:

"Health systems certainly are very difficult to define through hypothesis-driven research. The systems seem to change too quickly. I wonder sometimes whether the classic scientific methods of defining hypothesis, holding conditions stable and changing one variable, whether the training of many science folks engaged with health policies—that background, that training—is sometimes not useful in terms of understanding the descriptive methodologies and the quality improvement methodologies that tend to inform policy. You know people with a scientific background tend to look for that hypothesis during research and when it’s not there they say the material’s not useful. That’s a real problem. (CEO CANADA)"

### Sentiment in policy-maker interviews

**Figure 2:**
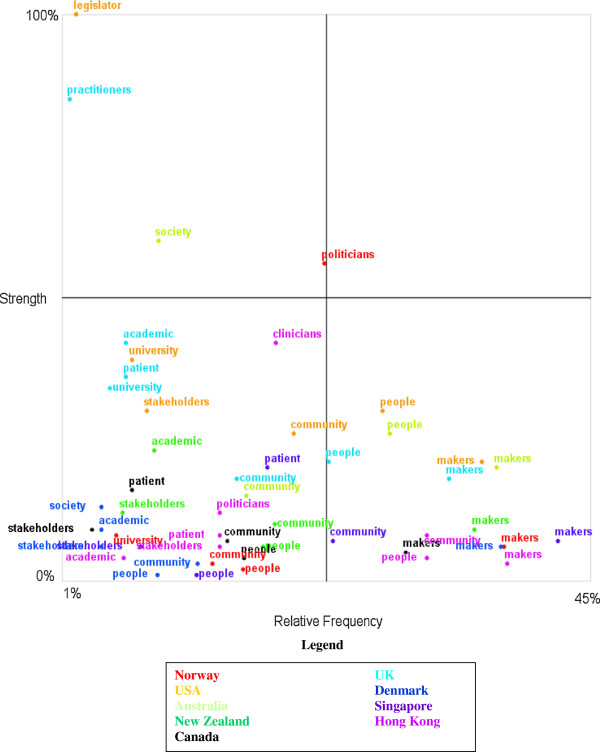
Quadrant map of references to ‘people types’ associated with sentiment terms in CEO interviews, by country.

Figure 2 provides the detail of all the terms used for people in the language of policy-makers associated with positive and negative sentiment words in this interview set. The relative frequencies or conditional probability of each term such as ‘scientists’ or ‘patient’ or ‘academic’ or ‘policy-maker’ being found with positive or negative sentiment words is mapped by grouping interviews by country (country groupings are for the sake of convenience only so that the quadrant will more clearly display differences between people type words in the language of policy-makers).

That is, Figure 2 represents the extent to which concepts about different kinds of people are found in the positive or negative language of CEOs grouped by country. The explanation that follows has been closely adapted from the software output [79]:

This is a high-level, visual chart displayed in a 'magic quadrant’ format. The axes are:

•Relative Frequency: a measure of the conditional probability of the concept, given the category (either positive or negative sentiment); for example, given we are looking at occurrences of either a positive or negative sentiment, how likely is it that the concept ‘academic’ is mentioned

•Strength: a measure of the conditional probability of the category (in this case either positive or negative sentiment) given the particular concept; for example, given we are looking at occurrences of the concept 'academic’, how often is it mentioned in either a positive or negative sentiment, i.e. the ‘strength’ of the association.

There are four pertinent areas to the quadrant: concepts in quadrant 1 (bottom left) are weak in terms of their frequency of use with sentiment associations and more likely to occur with negative terms while concepts in quadrant 4 (top right) are strong and more likely to co-occur with positive terms. The differing colours of the concepts denote their association with the particular country where the CEOs are located [79].

In other words, the y axis relates to frequency of association with sentiment words, while the x axis ‘splits’ those associations by whether the majority of instances of the concept are positive or negative. Concepts located above the x axis are majority positive associations, while concepts located below the x axis are majority negative associations. The word ‘majority’ is important here because in practice each concept must be placed in a way that accounts for what is generally true for that concept i.e. true when all instances of text blocks for that concept are considered. Rarely will a concept be exclusively positive or negative because for that to be true the concept must be uniformly referred to (negatively or positively) across all instances of that concept. There are exceptions: the ‘legislator’ concept was mentioned only about 1 out of a hundred times by USA CEOs but was positive 100% of the time (this is not a finding that suggests USA CEOs think highly of legislators, simply that their infrequent mentions of legislators in the language of sentiment were positive, which makes some sense in the context of the politically sensitive environments to which they are habituated).

Thus the quadrant describes four dimensions: more frequent (and either majority positive or majority negative) versus less frequent (and either majority positive or majority negative). For example, looking at the legend for Figure 2, the fact that the Singapore CEO’s use of the ‘policy-maker’ concept lies far along the × frequency axis (which extends to 45 out of a 100 times in the language of sentiment) suggests a relatively high frequency of sentiment association. That is, in a relatively greater number of times compared to other CEOs (36% to be precise when checking the data output) in the sentiment language this CEO used, was the ‘policy-maker’ concept used. This begs the question of what kind of sentiment language was used with the ‘policy-maker’ concept by this particular CEO. The y axis (which extends to 100 out of a 100 times in the language of sentiment) provides the answer: mostly negative. The data output confirms that in 7% or seven out of a hundred times in which the concept ‘policy-maker’ is used by the Singapore CEO in sentiment language is it associated with positive sentiment (all other sentiment associations are negative, which means the majority of associations for this concept are negative, and the concept therefore lies a long way down from the x axis).

Accordingly, the quadrant allows us to consider whether these interviews support the broad hypothesis that policy-makers use terms associated with academics more negatively than terms associated with other groups i.e. when asked to describe the features of research they find useful. It is worth noting that it does not measure the production of concepts in emotionally neutral language.

Care needs to be taken in interpreting Figure 2. For example, the fact that one policy-maker (a Norwegian CEO—see figure legend) used the concept word ‘politician’ more often in more positive language than other policy-makers, is not a finding that can be used to ‘determine’ that interviewee’s ‘true feelings’. There are many reasons that such an interviewee might produce more positive associations in an interview context, for example, a well-cultivated habit of self-preservation. However, viewed in aggregate, such a sentiment analysis can offer indicative evidence to help explore hypotheses such as ‘Policy-makers uniformly hold more negative views of researchers than they do of themselves when talking about policy-relevant research.’ Accordingly, the discussion that follows should be considered as an extension of our main analysis—as indicative supplementary evidence.

Figure 2 suggests that, when using this ‘language of the emotions’, policy-makers referred to themselves far more often than they did other groups. This is not really surprising given that they were being asked to focus on their own needs. However, there is no evidence of systematic differences in the degree to which references to policy-makers versus other groups such as academics or university communities were associated with positive or negative sentiments (i.e. when describing policy-relevant research methods as the study asked them to do). In fact, Figure 2 suggests some policy-makers did tend to refer to academics and university communities more positively than they did themselves, where such references occur. The language in these interviews works to normalise the view that policy-makers own the challenge of developing better research methodologies for policy i.e. that it is their challenge as much if not more than research communities. Yet there are no references to academics and university communities that are definitely positive, as for example, in the following example of the most positive reference to academics found in the interviews:

"So you have to sort of step down from the high academic horse and get into this everyday prioritising, discussions, you have to play a role in the newspapers, you have to play a role—you have to address, directly address policy-makers both on the political and administrative level, so your research is sort of playing to the context where decisions are made. If your research is not placed within that context the chances are that they won’t play any role when the decision is made; but if you’re able to place your research result into that context arena of decision-making then it might be influential on the eventual outcome of policy-making. (CEO DENMARK)"

Manual checks of sentiment-linked references to academics and university communities in these interviews versus those that were not sentiment linked were also undertaken using Leximancer. These suggested that a general lack of engagement with academic and university communities (not necessarily evidence per se) might explain the limited references to them in the ‘language of emotion’ of these policy-makers i.e. language that is apparently sentiment-linked. It appears from Figure 2 that, when discussing features of useful research, generally policy-makers in this study did not see a more positive role for themselves than they did for academics.

While Figure 2 suggests that the language of discussion about research practice is not a language that is generally positive, scrutiny of how these policy-makers used the word ‘community’, suggested they naturalised the idea of their own evidence-based commitment to their communities of interest. That is, they positioned themselves as having a key role in understanding and using information about local communities through the mechanism of authentic evidence. Negative sentiment was more about the ability of existing research practices to engage with the complexities of measuring community views and experiences than the communities themselves. Such language suggested that policy-makers’ struggled to find subtle and (technically and politically) robust methodologies of community-based research. In particular, policy-makers portrayed community-based research involving stakeholders as critical to authentic, democratic, evidence-based policy-making—almost normalising it as a contested site of evidence on which policies were won and lost. This was especially notable in the USA interviews:

"It really is an area with a lot of pitfalls and it’s an area I think where a researcher’s credibility really is put on the line, particularly when you’re asked to draw stakeholders together and get their points of view. It’s very easy to think of the people the researcher knows or has the ability to get in contact with as representative. I’ve seen a lot of effort lose credibility and the goodness that’s in the work, the truths that are there, not get put to use because it’s very easy for policy-makers who may disagree with the findings to discredit stakeholder-based work. I guess drawing the tent as broadly as possible would be, you know, an obvious bit of advice and probably touching base early on in the design with who the likely opponents or nay sayers will be and having them suggest people who need to be included. (CEO 4 USA)"

The role of expert opinion was little present in the ‘language of emotion’ of policy-makers in these interviews. However, there was at least one CEO who positively positioned research capacity-building among practitioners who were described as beginning with a clinical ‘research bias’ based on practical experience but ultimately offering the potential for research translation into healthcare benefits:

"It’s not just the training, it’s actually bringing on board on the clinical side practitioners with a research bias and encouraging them during their career development to be working alongside the scientists in all that they do—and endeavouring to influence and steer so that we can begin to see practical benefit. (CEO UK)"

## Discussion

Care must be taken not to infer that the structure and nature of this language dataset as found by our two-stage method is representative of the nature of the larger evidence-policy divide. At the same time, these interviews do not support simplistic definitions of policy-makers and researchers as coming from two irreconcilable worlds. It appears in these interviews at least that the worlds of policy and research are seen as separate but certainly not exclusive. Understanding their rich interface better is an important challenge for all those who want their research to better shape the health systems that practitioners and patients experience. Policy-makers in this study did not so much devalue research as distance research in ways that suggest they have their own language for legitimating its non-use—just as researchers do.

The view that the evidence-practice divide is also partly about the methodological limitations of research was also supported by these interviews. In this study, the needs for research into community health and system improvement were portrayed as complex and not well served by existing research evidence available to policy-makers. In short, the way policy-makers described the evidence-policy divide suggested not simply that they need technical quantitative research simply presented. It also suggested that they need technically and politically robust complexity-oriented evidence for local community health and service development.

‘Complexity-oriented research’ in these interviews can be defined as research that authentically and accurately captures the rich contexts in which policy-makers operate. This may be described as ‘community-based research’ where it involves an engagement with the local stakeholders important to policy-making—their views and experiences as part of ‘consensus-making’ or ‘consensus-finding’ for policy. This study does not offer evidence about the quality of such research, although our introduction does observe that the effort to capture contextual complexity is key in contemporary debates about the usefulness of research

Interestingly for those who have perpetuated the ‘two worlds’ view of the evidence-policy divide, policy-makers did not describe a more positive role for themselves than they did for academics in meeting such methodological challenges and addressing such evidence deficits. That they positioned community-based research as a vital evidence mechanism for policy is instructive. That such community-based research lies outside dominant definitions of high quality research for health exemplified by the CONSORT statement [80] may itself have worked to prevent development of excellence in local community and services-based research.

Such observations follow from the evidence of these interviews if we accept that there is some truth in how the CEOs understand the evidence-policy divide. However, we are conscious also that studies such as this one, asking the most senior health agency staff to describe the features of policy-relevant research, offer their findings in contexts where other triangulating evidence is very limited. For example, is it possible that all these interviewees have misunderstood the nature and purpose of quantitative and qualitative research? The involvement of CEOs from different countries, many with advanced research qualifications such as Ph.Ds, and their daily involvement with research evidence, makes it unlikely that they do not understand the fundamentals of research practice. At the same time, in a century in which many examples of quite ‘technical’ qualitative research also exist, the fact that policy-makers described qualitative research as having a popularist or non-technical nature offers support for the view that their ideas about research are just that. It is clear that they are only one kind of observer of the evidence-policy divide, albeit critical observers. More junior health agency staff more intimately involved in evidence-based project work may have offered different perspectives.

Notwithstanding, to shape the evidence-using behaviour of policy-makers, arguably we first need to understand how they perceive evidence—where this study has aimed to make its contribution. The fact that these CEOs have these kinds of views of research offers indicative evidence about the myths about research that researchers need to engage with if they are to persuade senior policy-makers of the value of their evidence. Even if these particular senior policy-makers are entirely wrong in their understandings of the true causes of the evidence-policy divide, this study suggests how their understandings may be working to perpetuate that divide. In practical terms in the health sciences, this may mean that researchers need to pay more attention to conveying the value of generalisable clinical quantitative methods—not only building excellence in applied community-based methods. For example, this study suggests that the work of colleagues such as Tunis developing pragmatic clinical trials [81] needs to be better conveyed to policy-makers who view RCTs very traditionally. The value of new developments such as ‘Comparative Effectiveness Research’ focussing on the policy-relevant question of ‘What works?’ [14] may also need to be better conveyed to policy-makers to help reinvigorate their ideas of research relevance.

Our final discussion points relate to our own methodological limitations and strengths. In relation to our sample, it is not clear how representative these policy-makers are of all health agency CEOs. The small numbers involved meant that the study focussed on aggregating viewpoints rather than examining regional variations in CEO perspectives. That is, we did not feel we could make conclusions about differences between countries or states in a context where we knew our sample was not representative of all state/provincial and national agency CEOs. Even if a representative sample was obtained we are uncertain that it would be accurate to treat CEOs as ‘data carriers’ of regional variations because many of them have international careers and their reference points often appeared to come from their experience in countries other than those whose health systems they administered at the time of the interviews. Accordingly we emphasise the value of the study as an aggregation of viewpoints—insights about a global social group we describe as ‘health agency CEOs’. We point to how the perspectives of health policy-makers at this level are rarely included in definitions of the research-policy-and-practice divide. The substantial resources we invested in locating and approaching them, and the general difficulty of accessing professionals at this level in health systems is perhaps part of the reason for their lack of visibility in the literature.

In relation to our analytic procedure, this aimed to combine the strengths of two ways of analysing language in order to help overcome the weaknesses of both. Leximancer offers an approach to content analysis of qualitative data but as a machine-driven, content-focussed method does not offer a theoretical approach to understanding language that can also help analyse its subtle nuances. Critical discourse analysis offers a well-established theoretical framework for analysing how meaning works in language but has been criticised for a perceived lack of empirical rigour and reliability [76]. Rather than arguing for any method, we believe that an openness to the possibilities of combining different methods may help qualitative research better satisfy the needs of the health sciences.

## Conclusions

We conclude that these particular interviews do not support simplistic definitions of policy-makers and researchers as coming from two irreconcilable worlds. The policy-makers interviewed for this study value qualitative and quantitative research. However, they suggest that to be policy-relevant health research may need to focus on building complexity-oriented research methods for local community health and service development. Researchers also need to better explain and develop the policy-relevance of large statistical generalisable research designs. Policy-makers and public health researchers wanting to serve local community needs may need to be more proactive about questioning whether the dominant definitions of research quality and the research funding levers that drive university research production are appropriately inclusive of excellence in such policy-relevant research.

## Competing interests

The authors declare that they have no competing interests.

## Authors’ contributions

EB designed the study and interviewed the health policy-makers and produced the first draft of the paper, including the critical discourse analysis. BS provided valuable input on research design and the use of Leximancer. BS offered critical input on the study findings, i.e. both the use of Leximancer and the critical discourse analysis, as he was not involved in the interviews. Both authors read and approved the final manuscript.

## Authors’ information

EB is deputy director and associate professor at the University Department of Rural Health, University of Tasmania. She has published over 80 academic and applied publications with a particular focus on complex multi-disciplinary policy development challenges. Her work in research methods for health policy-makers is represented in her books *Research for Health Policy* (Oxford University Press 2010) and *Translational Research for Primary Healthcare* (Nova Science, 2012). Prior to her employment in 2004 in rural health she was a policy research manager in the Queensland government leading a diverse multidisciplinary research team.

BS is a general practitioner at the Huon Valley Health Centre, Tasmania, and also clinical Senior Lecturer at the Discipline of General Practice, University of Tasmania, and Affiliate Research Fellow at the Discipline of General Practice, The University of Adelaide. He gained a PhD from Leipzig University in pediatric immunology and qualified as a general practitioner in the United Kingdom during which time he published on pediatric immunology and was lead author of a clinical book on medical classifications. He has practiced in diverse contexts, including emergency medicine in South Africa and mental health and obstetrics in rural practice in the United Kingdom.

## Pre-publication history

The pre-publication history for this paper can be accessed here:

http://www.biomedcentral.com/1471-2458/12/932/prepub
